# NADPH oxidases: Current aspects and tools

**DOI:** 10.1016/j.redox.2020.101512

**Published:** 2020-05-23

**Authors:** Katrin Schröder

**Affiliations:** Institut für Kardiovaskuläre Physiologie, Fachbereich Medizin der Goethe-Universität, Theodor-Stern Kai 7, 60590, Frankfurt, Germany

## Abstract

Reactive oxygen species (ROS) have been shown or at least suggested to play an essential role for cellular signaling as second messengers. NADPH oxidases represent a source of controlled ROS formation. Accordingly, understanding the role of individual NADPH oxidases bears potential to interfere with intracellular signaling cascades without disturbing the signaling itself. Many tools have been developed to study or inhibit the functions and roles of the NADPH oxidases. This short review summarizes diseases, potentially associated with NADPH oxidases, genetically modified animals, and inhibitors.

## Graphical abstract

**Figure:**
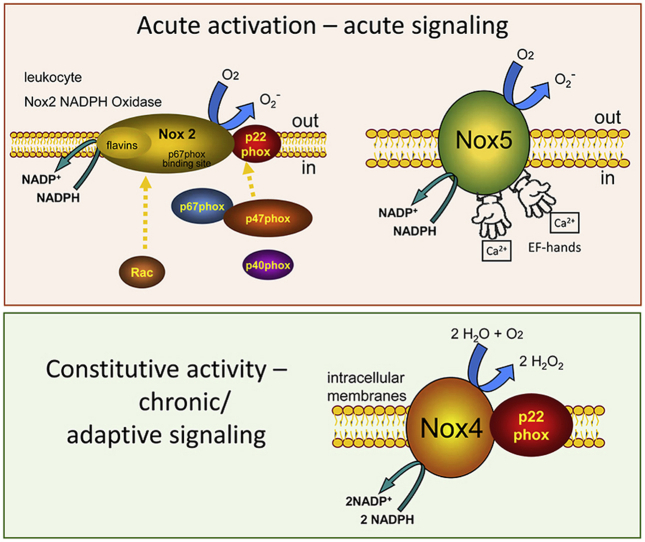

## Main

**Fig. 1:**
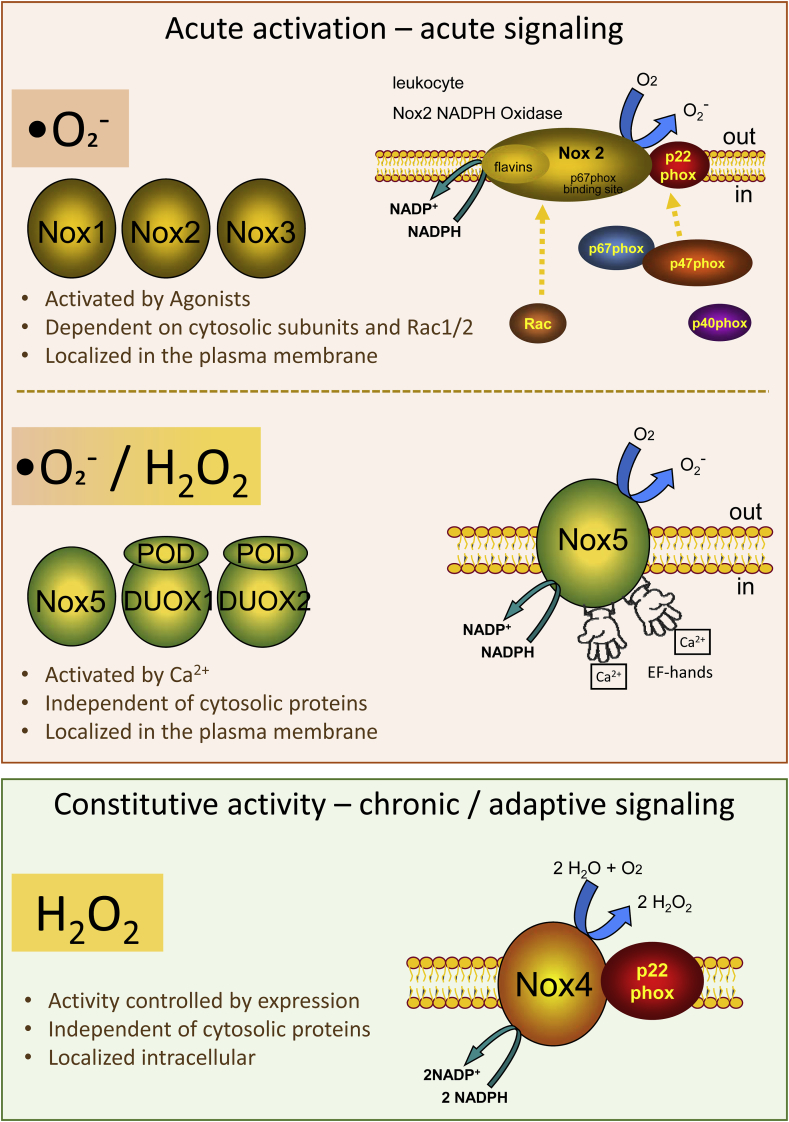
**Scheme and classification of the members of the NADPH oxidase family.** NADPH oxidases can be classified into three groups according to their mode of activation. Nox1-3 are activatable via the assembly of cytosolic subunits and produce •O_2_ˉ. Nox5 and the Duoxes can be activated by Ca^2+^ and produce •O_2_ˉ or H_2_O_2_. The single member of group three is Nox4, which produces H_2_O_2_ in a direct manner independent from cytosolic subunits. Further explanations can be found in the text.

The family of NADPH oxidases consists of 7 members. Those are Nox1 through 5 and Duox1 and 2. All NADPH oxidases are able to transfer electrons across biological membranes. Those electrons are provided by NADPH. While passing the membrane through the Nox subunit, electrons are transferred onto molecular oxygen to generate superoxide anions (•O_2_ˉ). •O_2_ˉ can be released unmodified or protonated and reduced to form H_2_O_2_. Despite this, NADPH oxidases differ in their mode of activity. Both together allow for a systematic classification of the individual members of the family into three groups (Fig. 1).

The acutely activatable NADPH oxidases Nox1, Nox2, and Nox3 can be pooled into group 1. The appropriate complex consists of the name-giving Nox subunit and the smaller p22phox, which stabilizes the Nox protein. Nox1-3 depend on the association of the membrane bound subunits with cytosolic proteins. The interested reader is referred to Ref. [1] for detailed information concerning the cytosolic subunits of Nox1-3. Shortly: The cytosolic components are organizers (p47pox or NoxO1) and activators (p67phox or NoxA1). The organizer proteins p47phox or NoxO1, facilitate the assembling of the other cytosolic components into the full NADPH oxidase complex. P47phox contains an autoinhibitory region (AIR). Upon phosphorylation, this AIR gets inactivated and p47phox translocates to the membrane and binds p22phox. In contrast to p47phox, its homologue NoxO1 has no AIR and shows constitutive activity, which can be modified by phosphorylation. Accordingly, phosphorylation of the organizers facilitates acute cytokine-induced ROS formation by Nox1-3. Importantly, although in overexpressing systems the cytosolic subunits can substitute for each other, this does not occur in vivo, as their expression is cell specific [2,3]. Therefore, the absence of p47phox is not counterbalanced by an elevated expression of NoxO1 and vice versa. In leucocytes, an additional subunit, p40phox, is needed for the full complex to be associated. Additionally the non-NADPH oxidase specific G-protein Rac binds to the NADPH oxidase complex in order to activate the formation of superoxide radical anions (•O_2_¯) by the members of group 1.

The second group of NADPH oxidases consists of the Ca^2+^ activated Nox5, DUOX1 and DUOX2. These NADPH oxidases are independent of cytosolic factors but instead have EF-hands that facilitate the Ca^2+^ sensing. While Nox5 produces mainly •O_2_¯, DUOX1 and 2 produce both, •O_2_¯, as well as H_2_O_2_ probably with the aid of their peroxidase domain (POD). Both Duoxes require the maturation factors DuoxA1 and 2 for their activity.

The sole member of the third group of NADPH oxidases is Nox4. Like Nox1-3, Nox4 is stabilized by and associated with p22phox. Despite from that Nox4 does not require any further cytosolic subunit and therefore is constitutively active. Due to a special loop in its structure Nox4 is capable to restrain single reduced •O_2_¯ and reduce it further to H_2_O_2_ [4].

Over and above their different mode of action, NADPH oxidases also have individual intracellular localization and tissue specific expression patterns [5]. Expression and activity of NADPH oxidases are tightly controlled which enables the individual members of the family to interfere with numerous paths of signal transduction. Those include oxidation of phosphatases or kinases [6,7]. According to their complex role in regulation of cellular signaling, individual members of the family have been assigned for a number of diverse diseases in humans. Some of those are summarized in Table 1.

**Table 1:**
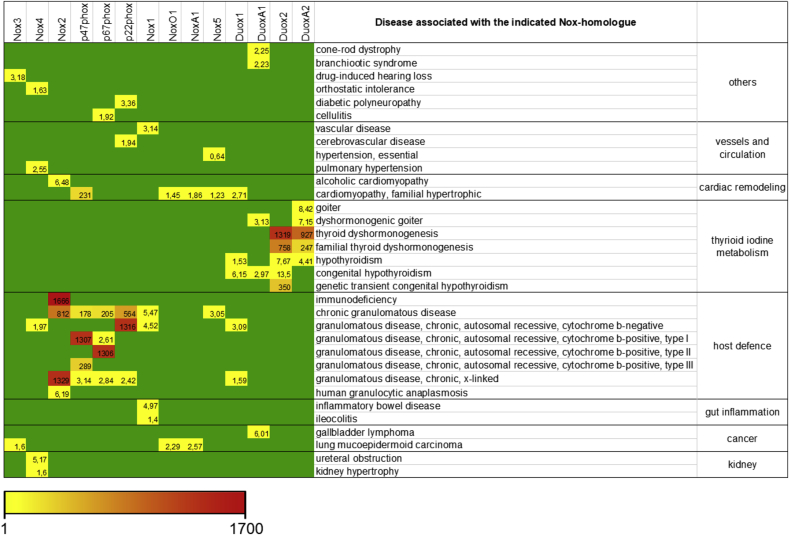
List of some diseases associated with NADPH oxidases.

All diseases listed were obtained through www.gencards.com. The analysis shows the results in the disease section of each gene in GeneCards, which is based on the MalaCard website and score. The MalaCards score ranks diseases by how closely they are associated with the gene, factoring in the relative reliability of the sources that associate them. Green indicates no relevant entry. Relevant entries are represented by numbers and the color scale indicated.

Table 1 shows basically two clusters of diseases associated with NADPH oxidases: chronic granulomatous disease and thyroid hormone production. This reflects the so far identified physiological role of the parties thereto. Nox2 and its associates p47phox and p67phox are needed for a proper fist line host defense, known as “the oxidative burst”. Accordingly, CGD (chronic granulomatous disease), a disease with inproper function or absence of one of the components of the Nox2 complex, represents with frequent infections by fungi and bacteria [8]. Duox2s physiological function is to oxidize iodine for its incorporation into the thyroid hormone. In case Duox2 or its maturation partner DuoxA2 is not present, the formation of the hormone is reduced and all kind of subsequent diseases phenotypes such as goiter and hypothyroidism occur [9]. Besides those clear cases of a physiological function of NADPH oxidases, that lead to a more or less defined and namable disease, many functions of the members of the family are unclear. Accordingly, no diseases have been identified with are solely based on the function or dysfunction of Nox1, Nox3, Nox5 or Duox1. Nevertheless, they appear to play a role in prevention or as contributors to several diseases, where their exact role often remains to be defined. In conclusion, NADPH oxidases obviously rather modulate (the development of) than cause a disease. Most literature indicates a detrimental role of NADPH oxidases in human diseases. However, beneficial roles of NADPH oxidases and ROS formation become more and more clear. As an example, Nox4 plays a role in angiogenesis, prevents bone loss upon estrogen depletion and protects from atherosclerosis [[10], [11], [12]].

Accordingly, research of the role of specific NADPH oxidases is needed to obtain a deeper understanding of their physiological roles. Tools such as knock out models or specific inhibitors have been developed. An overview of currently available animals with knock out, knock in and loss of function mutants of NADPH oxidases is provided in Table 2. This table however, might be incomplete and just provides an overview. Besides full animal approaches, the CrispR/Cas9 method opens a variety of possibilities to study the role of individual subunits of the NADPH oxidase complexes on cellular level. This approach has been successfully used, for example in Hek293 [13] and human HCT116 colon cancer cells [14].

**Table 2:** List of some animal models for NADPH oxidase research.

Target	Tool		
	knock out animal	knock in animal	Loss of function mutants
**Nox1**	Mouse [15] Mouse floxed [16] Zebrafish [17]		
**Nox2**	Mouse full ko [18] Mouse floxed [19] Zebrafish [17]		
**Nox3**			Mouse [20,21] Mouse [22]
**Nox4**	Mouse full and floxed [23]		
**Nox5**	Rabbit [24,25] Zebrafish [17]	Mouse [25,26]	
**DUOX1**	Zebrafish [27] Mouse [28]		
**DUOX2**			Mouse [29]
**DuoxA1&2**	Mouse floxed [30]		
**p22phox**	Mouse floxed [31]		Mouse [32] mouse [33,34] + tyrosinase(−/−) rat [35]
**p47phox**	Mouse [36]	Mouse [37]	Rat [38] Mouse [39]
**NoxO1**	Mouse [40]		
**p67phox**	Mouse [41]		Rat [42]
**NoxA1**	Mouse floxed [43]		Mouse [44]
**p40phox**	Mouse [45]		Mouse [46]

Additionally, a brief collection of available inhibitors was added here (Table 3). For a detailed overview on NADPH oxidase inhibitors, the reader is referred to Ref. [47]. In that specific publication, the authors highlight the evolution as well as the limitations of Nox-inhibitors, antioxidants and other related compounds.

**Table 3:** Inhibitor peptides and small molecules that act as NADPH oxidase inhibitors.

Target	Inhibitor peptide	Pharmacological inhibitor
**Nox1**	NoxA1ds (mimics a putative activation domain of NoxA1 and p67phox amino acids 199–210 in the FAD with substitution of Y199 by alanine 196 EPVDALGKAKV-CONH2 [48]	ML171 [49] GKT136901 and GKT137831 [50,51]
**Nox2**	Endogenous PR-39 (RRR PRP PYL PRP RPP PFF PPR LPP RIP PGF PPR FPP RFP) [52] several peptides (peptide walking) [53] B-loop peptide of Nox2 that binds to p47phox: C^85^SRVRRQL^93^ [54] → Nox2ds-tat [55] works in vitro and in vivo (specifically inhibits the interaction of Nox2 and p47phox [56])	GSK2795039 [57] CYR5099 [58] Bridged tetrahydroisoquinolines: CPP11G and CPP11H [59] Perhexiline and Suramin (cell impermeable) [60]
**Nox4**		GLX7013114 [61] GKT137831 [50] GKT137928 [62] ACD084 [63] Rosmarinic acid [64]
**Nox5**	peptides pep1 and pep3 containing a KDSIT sequence at the c-terminus (D637−G661 + Y and R621−T660) [65]	
**Duox1 and Duox2**	S–P-Re-J-L, wherein Re is a reactive electrophile and J is G or P [66]	Acrolein [67]

Besides specific inhibitors, many global inhibitors for NADPH oxidases (or flavoproteins in general) and antioxidants are used. Those include diphenylene iodonium (DPI), apocynin, diapocynin and ebselen [68]. Some derivatives of the antioxidant ebselen, such as JM-77b, had a selectivity for Nox2 over Nox1, Nox4 and Nox5 [69]. This however does not mean, ebselen derivatives are specific Nox2 inhibitors. Especially in the light of the fact that ebselens are reported to display glutathione peroxidase-like activity [70].

In contrast, potential specific inhibitors often have been proved to be not specific or display off-target effects. The best investigated NADPH oxidase, Nox2, may serve as an example: Formerly known Nox2 inhibitors such as VAS2870 [71,72] and VAS3947 [73] did not fulfill their assigned roles as specific inhibitors. Both have been identified to exhibit off-target effects through thiol alkylation and inhibition of mitochondrial respiration and cytotoxicity [74,75]. Substances like celastrol inhibit Nox1, Nox2, Nox4 and Nox5, as it interferes with the binding of the proline rich region of p22phox to the tandem SH3 domain of p47phox and NoxO1 [76]. Alike, the PR-39 peptide binds other SH3-containing proteins, such as p130Cas and PI3Kp85α [77,78]. Recently, it was documented that also the Nox1/Nox4 inhibitors GKT136901 and 137831 are in fact non-specific [68,79]. Additionally, the Nox1 inhibitor ML-171 was also shown to be unspecific [80]. It appears that specificity of the inhibitors targeting a common domain in NADPH oxidases can be impeached. For further reading on how inhibitors work and fail the reader is referred to the work of Vincent Jaquet (Geneva) and Harald Schmidt (Maastricht).

## Concluding remarks

Understanding the role of individual NADPH oxidases bears potential to interfere on a modulatory basis with intracellular signaling cascades. Within the last years, the collection of tools to analyze and target NADPH oxidases increased constantly. Therefore, it is important to provide an overview from time to time. This short review summarizes diseases potentially associated with NADPH oxidases, genetically modified animals, and inhibitors for some members of the family. Most references either point to a location, where to get the animals or to the first description of the animal or inhibitor. This should enable the reader to find a way to his/her tool of interest.

## Funding

This work was supported by grants from the 10.13039/501100001659Deutsche Forschungsgemeinschaft (DFG) (to KS SCHR1241/1-1, SFB815/TP1, SFB834/TPA2).

## Declaration of competing interest

The author declares no conflict of interest.
